# Perceptions of Good-Quality Antenatal Care and Birthing Services among Postpartum Women in Nepal

**DOI:** 10.3390/ijerph18136876

**Published:** 2021-06-26

**Authors:** Sushma Rajbanshi, Mohd Noor Norhayati, Nik Hussain Nik Hazlina

**Affiliations:** 1 Women’s Health Development Unit, School of Medical Sciences, Health Campus, Universiti Sains Malaysia, Kelantan 16150, Malaysia; sushmarajbanshi@yahoo.com (S.R.); hazlinakck@usm.my (N.H.N.H.); 2 Department of Family Medicine, School of Medical Sciences, Health Campus, Universiti Sains Malaysia, Kelantan 16150, Malaysia

**Keywords:** quality of care, users’ perspectives, good antenatal care, good birthing services, reproductive rights, patient-centered care, Nepal

## Abstract

Patient complaints and dissatisfaction should be taken seriously and used as an opportunity to provide acceptable services. Mounting evidence shows that the perception of the quality of healthcare services impacts health-seeking behaviors. This study explores the perceptions of good-quality antenatal and birthing services among postpartum women. A qualitative study using phenomenological inquiry was conducted in the Morang district, Nepal. The study participants were postpartum women with at least one high-risk factor who refused the referral hospital’s birth advice. A total of 14 women were purposively selected and interviewed in-depth. NVivo 12 Plus software was used for systematic coding, and thematic analysis was performed manually. Three themes emerged: (i) women’s opinions and satisfactory factors of health services, (ii) expectations of the health facility and staff, and (iii) a lack of suggestions to improve the quality of care. Women did not have many expectations from the healthcare facility or the healthcare providers and could not express what good quality of care meant for them. Women from low socioeconomic status and marginalized ethnicities lack knowledge of their basic reproductive rights. These women judge the quality of care in terms of staff interpersonal behavior and personal experiences. Women will not demand quality services if they lack an understanding of their basic health rights.

## 1. Introduction

The availability of maternal health services at a health facility does not always guarantee their access and use by women [1]. Perceptions about poor-quality health care and experiences can discourage patients from using the available services and delay seeking care [1,2,3]. There is growing evidence that the perceived quality of care services has a greater influence on patients’ behavior, and the quality of care should be monitored based on their perceptions [2,4,5]. However, quality is not easy to define or measure [1]. The quality of care is viewed subjectively by individual patients [6]. Suppose a woman is unhappy with the quality of services and disrespectful treatment she receives. In that case, it does not matter how highly competent the clinical staff is; she may not use the services [1]. Therefore, assessing patients’ perspectives of the quality of care gives them a voice to make services more responsive to their needs and lead to better health outcomes [1,7].

All women want facility staff to provide “good” care. However, “good” care has multiple connotations [8]. Clients were found to be willing to pay for private services and travel far if they perceived that a good quality of care is offered [9]. However, healthcare professionals measure the quality of care in terms of process rather than outcome [10], while patients measure it based on a combination of experiences, expectations, and perceptions [11,12]. In addition, women form their perceptions according to friends’ and relatives’ experiences, myths, and societal values [12].

High maternal deaths are still a concern in Nepal, where the current maternal mortality ratio is 239 per 100,000 live births in 2016 [13]. Nepal aims to reduce the maternal mortality ratio to less than 70 per 100,000 live births by 2030, in line with sustainable development goals. However, nearly one-fifth of pregnant women (22.2%) discontinue their four antenatal visits, and a similar percentage of pregnant women (22.5%) avoid institutional births [14]. In Nepal, 52% of women are married by 18 years of age, and the adolescent birth rate is 63% [13,14]. Still, inequitable access and utilization of maternal health services exist among poorer women [15]. Caste and ethnicity are important drivers of social hierarchy and access to care in Nepal [16]. Access and utilization of healthcare services have attracted increased attention lately.

One factor that hinders the acceptance of referral advice is the perceived quality of care at the hospitals [17,18]. Women will have their reasons for denying receiving services from the health facility, which need to be explored. Disrespectful treatment of laboring women is one of the reasons [19]. About one-third of women have reported experiencing disrespectful maternity care services in Nepal [20]. The quality of care in maternity services has received inadequate attention [1]. Despite this, quality of care is considered a key component of the right to health and the route to equity and dignity for women and children [21]. Quality of care can be measured from the provision of care provided from within the institution and as experienced by users [1]; the latter is explored in this study. This study attempts to explore women’s perceptions of good-quality antenatal care (ANC) and birthing services. Gaining an understanding of women’s perceptions of the quality of care may lead to an improvement in public policies and result in better care for women.

## 2. Materials and Methods

### 2.1. Study Design

This study applied the qualitative method and used a phenomenological design. The study was conducted in the Morang district of Nepal from November 2019 until April 2020. Postpartum women of any age who were Morang residents had at least one high-risk factor in pregnancy and refused referral birth at a tertiary hospital were enrolled. The Presence of any high-risk factors, such as high blood pressure, pre-eclampsia, pregnant women with medical problems receiving hospital treatment, high fever for more than seven days, etc., that were listed as red or yellow codes based on the Malaysian risk stratification approach was used to select the participants [22]. The purposive sampling method was used. The sample size was selected following the concept of saturation. The data generation was stopped when the collection of new data did not shed further light on the issue of interest [23]. A total of fourteen postpartum women were interviewed in-depth.

### 2.2. Study Settings

The Morang district is situated in the eastern region of Nepal. It is the second-most densely populated district, with a population of 1,073,307 [24]. The estimated number of expected pregnancies in the Morang district is 27,799 per year [24]. The institutional birth rate of Province 1, where the Morang district is located, is 62% [24]. In Morang, 14.4% of pregnancies were high-risk pregnancies in 2020 [25]. The population living in the Morang district is heterogeneous and diverse. The rural population coverage is 78% [26]. Disparities in access to healthcare services exist in Nepal based on ethnicity, especially among underserved populations [27]. Women from the underserved groups are lower utilizers of essential healthcare services compared to affluent groups who are more likely to receive better treatment and access [27]. Women from the marginalized community have diminished autonomy and are financially dependent on family members to access institutional births. Furthermore, multiple sociocultural factors hinder them from accessing care [28].

### 2.3. Sample and Recruitment

Postpartum women of the Morang district comprised the study population. Those living in the periphery of birthing centers in the Morang district were the targeted population. The study participants were postpartum women who met the inclusion criteria of a high-risk factor in pregnancy and refused the referral hospital’s birth advice. Details of this sample selection are mentioned in another study [28]. Postpartum women with either red or yellow codes based on risk factors listed on the Malaysian risk stratification approach [22] were identified by healthcare providers or the birthing center’s records. Once identified, the eligible postpartum women were approached in their respective community with the help of female community health volunteers. Women were interviewed at home and at the health facility, depending on participant’s time and convenience. Women were approached, informed about the study purpose and objectives, and invited to participate. The informed consent was read to the illiterate participants and written informed consent was obtained for adult participants, whereas an assent form was obtained for minor participants. The data generation process was performed following the Helsinki Declaration’s guidelines and regulations. Participants’ identity, anonymity, and confidentiality were maintained. Participants were well informed, and their permission was obtained for future publications. Oral consent was obtained to use an audiotape recorder. Women in the community mainly belonged to the minority ethnicity (Santhal, Muslim, and Dalits) and marginalized community living in proximity to rural areas of the Morang district. Three of the young participants’ mothers accompanied them during the interview. The majority of women were found practicing home births where interviews were taken. The birthing centers’ staff were asked about the most disadvantaged community locality nearby to find eligible participants. There were 23 birthing centers in the Morang district. It was challenging to find the women meeting our inclusion criteria. Out of 23 birthing centers, all interviewed women were located from six birthing centers. The participants’ final agreement was taken verbally for information that was provided by the mother. Initially, participants’ relatives would not leave the site of the interview. During this period, participants’ social background was asked, and later they were asked to leave for privacy reasons.

### 2.4. Data Generation

A semistructured interview guide was prepared. Broad topics and questions based on the study objectives were listed. The prompts and sequences of the checklist were not rigid [29], and the interviews were conducted in the Nepali language. The principal investigator conducted in-depth interviews with eligible women. Each interview lasted for a maximum of 45 min. Where necessary, female community health volunteers were used as translators if the local dialect in use was one other than Nepali. All interviews were recorded, so the essence of participants’ responses was not lost during translation. The interview started with sociodemographic information, followed by specific questions according to the study objectives. Brief sociodemographic information was taken at the beginning of the study for ice-breaking purposes. The recruitment of participants continued until data saturation was achieved. Data saturation was considered reached when the gathered data reached the point of diminishing returns when nothing new was being added, and there was a redundancy of information [30].

### 2.5. Data Analysis

The audiotaped recordings were transcribed and translated into English. The transcripts were checked for accuracy against the audio recordings. The verbatim transcripts were analyzed using computer-aided qualitative data analysis software (NVivo version 12 Plus). The principal investigator independently worked on the transcripts. Appropriate concept fitting codes were generated as suggested by the data for a few transcripts. The generated codes were validated by coauthors, which were subsequently applied to the remaining transcripts. New information was continually compared with previously coded data and their coding [30]. The scripts were first systematically coded, grouped into categories/subcategories, and finally grouped into themes [31]. Thematic data analysis was used to interpret the themes and patterns and prepare the manuscript (including relevant study participants’ quotations). Throughout the coding, the authors periodically discussed and revised the generation of codes, themes, and subthemes. The coding process was done using an inductive approach, whereas a deductive approach was used during themes generation.

## 3. Findings

### 3.1. Characteristics of Participants

Fourteen women were interviewed in-depth. The mean (SD) age of participants was 19.92 (3.71) years. Approximately 75% of participants were ≤18 years old. Among them, two were 17 years of age. The majority of participants belonged to the lower ethnic groups, i.e., Muslim, Santhal, Dalit, and Janajati. All women were engaged in home duties, and their husbands/partners worked in factories (n = 9, 64.3%), sales (n = 3, 21.4%), or farming (n = 2, 14.3%). Nearly two-fifths of the participants’ husbands lived away from home or abroad for work. Regarding women’s education, 21.4% had no formal education, 71.5% had primary and secondary education, and 7.1% had tertiary education. Except for three (21.4%), the rest of the participants lived in a joint family. Three of the fourteen women (21.4%) were unmarried but living at their partner’s house. A total of 57.1% of women had less than four antenatal visits, and a similar percentage were nullipara and gave institutional births.

Three major themes emerged by analyzing the 14 transcripts: (i) women’s opinions and satisfactory factors of health services, (ii) expectations of the health facility and staff, and (iii) a lack of suggestions to improve the quality of care (Table 1).

### 3.2. Theme 1. Women’s Opinions and Satisfactory Factors of Health Services

#### 3.2.1. Subtheme 1.1 Satisfaction with Existing Health Services

The recipients of health services play a central role in the assessment of the quality of care. Our participants were satisfied with how the healthcare providers communicated and treated them and their overall experience of giving birth at the birthing center. They were generally satisfied with the waiting time, check-up duration, patient flow, cleanliness, and medicine availability (free iron-folic acid tablets and anthelmintics).

*Good means everything is good … check-up was also done properly, everything, medicines were also provided, enough medicines.*

*(P007, 23 years, Madhesi)*

The average waiting time was approximately 10 to 30 min after registration, and the check-up duration was 5 to 10 min. The longest waiting duration mentioned was about two hours. Except for a few, the majority of participants did not complain about the waiting duration. All participants said that they considered the consultation time for antenatal check-up as “good”, except for one participant who mentioned that it was longer and, if possible, should be shortened.

For some participants, ANC was the time for them to socialize with their friends and relatives, where they enjoyed walking to the health facility and talking while they waited. A few women mentioned avoiding an ANC if there were no other companions to go along with them.

Video X-ray was a common terminology used for ultrasonography. In general, women had the idea that they had to do ultrasonography during pregnancy. Some went to private clinics for ultrasonography on their own, skipping their regular ANC visits because they thought that the healthcare providers would ask them to do it more than once. There was no consistency in the number of times the ultrasonography was done among the participants.

*Now, from five months of gestation, one should do ultrasonography, isn’t it? It has to be done three times. Repeating the same ultrasonography three times? That’s why we did it only once in the eighth month.*

*(P009, 17 years, Santhal)*

The majority of participants mentioned that the health facility was clean. Some said the cleanliness was good; a few mentioned it was fine. Only one participant, who belonged to an advantaged ethnic group and was educated up to a higher secondary level, reported that the health facility was dirty.

#### 3.2.2. Subtheme 1.2 Preferred Place of Birth and Less Concern about Health Issues

Participants had used both public health facilities and private clinics equally. Compared to the public health facilities, women who had utilized private services preferred these services. The main reasons for using private services for ANC were their quick service, renowned and experienced doctors, and flexible opening times outside office hours. The availability of laboratory services was another reason because most of the public health facilities lack this service.

Nine out of fourteen participants preferred to give home birth, which was encouraged and expected by their family members. Women preferred and trusted well-staffed and -equipped private and public hospitals for giving birth in the case of complications. The recommendations of female relatives based on their personal experiences were highly valued.

*All my seven children were born at home without any assistance. My mother-in-law is a traditional birth attendant. I didn’t even let her assist me during birth.*

*(P006, 30 years, Muslim)*

The majority of participants did not remember their weight or blood pressure, and they could not precisely explain what health problems they faced during pregnancy. Participants were not inquisitive enough to question the healthcare provider about their health or their fetus’ health.

*I don’t know how much my blood pressure was. I usually don’t memorize these things. I didn’t ask if my pressure was high or low. I didn’t realize I should have asked.*

*(P001, 21 years, Madhesi)*

Information regarding services intended to promote institutional births, such as cash incentive schemes, had not reached our participants. Women appreciated receiving transport incentive money worth NPR 1000 (USD 8.8) and free clothes for the mother and baby after birth at the birthing centers. Participants did not anticipate receiving free clothes, bed nets, and money and were happy to receive them. Some local government offices had added additional money to the existing government scheme to encourage institutional birth at the local birthing center.

### 3.3. Theme 2. Expectations of the Health Facility and Staff

#### 3.3.1. Subtheme 2.1. Expectations of Health Staff

Women did not explicitly mention what kind of staff behavior they expected. However, our participants frequently reported that during ANC or at birth, they appreciated healthcare providers’ friendly and open behavior. Participants liked it when the healthcare providers told them the dos and don’ts in pregnancy and explained them in detail. Some participants mentioned that they liked it when the staff asked them to visit again for follow-up and encouraged them to adhere.

Although the participants did not express it, reading between the lines, it was clear that familiar staff made them feel at ease at the health facility. They build a trusting relationship with these healthcare providers from the nearby birthing centers.

Participants who gave birth at the birthing center appreciated the staff coming to check on them after birth. For one participant, the staff came to check on her twice during her overnight stay, but she did not show any sign of anger or dissatisfaction; she was happy that they came to check on her.

During the births at the birthing center, a few women mentioned that some components of ANC were missed, such as one participant recalling that she was not asked to do a blood test during her ANC. She had done ultrasonography and a urine test but not blood grouping. Hence, she was scolded during the birthing time for not knowing her blood group. The other was a young participant. Staff at the health facility did not tell her the expected date of birth. When mild labor pain started, she stayed at home because it was not yet nine months, according to her calculation. She was unsure about whether to inform her husband. Through all this dilemma, the baby was born at home. Lack of counseling on family planning methods among young participants was another component missed by the healthcare providers.

#### 3.3.2. Subtheme 2.2. Expected Change in Health Services and Facilities

Women expressed that it would be such a relief for them if the ultrasonography and laboratory tests were available at the same facility. It would alleviate the need to walk or travel out of the village to get tested and save money. Some women fear that while traveling, there could be a risk to their pregnancy.

Participants mentioned that aside from giving free folic acid tablets and a physical check-up, the ANC service had nothing more to offer. The majority of women were dissatisfied because calcium supplements were not free. Calcium supplements were unaffordable or not available in the village; therefore, some women did not consume them while pregnant.

*The health post doesn’t provide calcium and women usually don’t buy calcium. The healthcare providers don’t understand where women will bring money. Calcium is prescribed to all; no one consumes it.*

*(P005, 25 years, Dalit)*

Although an ambulance service was available, its charge was considered unaffordable. A government-subsidized ambulance service rate was preferred and also suggested. Women and their family members find it inconvenient that there was only one bed at the birthing center and no beds for their waiting relatives. They complained that even though they wanted to stay overnight, the healthcare providers would send them back because there was only one bed. Women showed concern about who would receive service if two birthing women arrived at the same time. During summer, women expect bed nets because of mosquitoes. In winter, they suggest at least a heater at the birthing center.

*I said that let’s keep her overnight at the health facility, but it was said … all night staying here will be cold, “your daughter is fine” it will be cold here at health post.*

*(Mother of P004, 18 years, Muslim)*

Surprisingly, most participants mentioned that they did not have to use the toilet at the health facility or drink water. Participants laughed and answered that they use the toilet at home or drink enough water before leaving home for the health facility. Even the participants who mentioned that it took them two to three hours to arrive at the ANC service replied they had not used the toilets. The actual reasons for this behavior could not be explored because the participants kept laughing and did not answer.

*I have not gone to the toilet there. [started to laugh] … I have not drunk water there … I drink from home … [laughter again] … [still laughing].*

*(P002, 17 years, Muslim)*

Participants gave a mixed response to the crowding in health facilities. They understood that they had to wait before their turn. However, all the participants mentioned that they want services to be quicker. Sometimes, women were turned away to private clinics due to an overcrowded health facility, as women had household responsibilities to attend. This usually happened at the facility where children’s vaccinations and antitetanus toxoid vaccination days were merged with the antenatal check-up.

*It takes longer because the same day they immunize the children and check pregnant women. If I went for a check-up at 10 am, then I will return only around 1 pm.*

*(P0013, 19 years, Janajati)*

### 3.4. Theme 3. Lack of Suggestions to Improve the Quality of Care

#### 3.4.1. Subtheme 3.1. Cannot Express the Requirements of Good-Quality Services

Most participants mentioned that they find the health facility’s services good or very good (or in their language “badiyaan”) for both ANC and birthing services. Probing further into what was good, the majority of participants were unable to express themselves properly. For one participant, good service meant:

*Now, just like … the healthcare provider will explain the things that I don’t know and provide medicines … when and at what time to take … tell me things I didn’t know, … so I find it very good.*

*(P0012, 18 years, Janajati)*

The majority of participants expressed that they had no idea what needed to be improved to enhance the quality of existing ANC and birthing services. They said that “the healthcare providers would know better” than them about what to improve. Participants went completely silent or smiled, and their answers were “did not know much”, “what to say”, and “healthcare providers would know better”.

Participants did not mention that they need to be checked by the same healthcare provider every time. Sometimes, knowing that the same healthcare provider would be there to ask follow-up questions results in a hindrance. At other times, knowing the healthcare provider personally helped women approach them in an emergency, especially during labor onset at night.

*The health facility was already closed at 10 pm. Then my aunties went and called the XXX sister. There she lives nearby*.

*(P001, 21 years, Madhesi)*

#### 3.4.2. Subtheme 3.2. Lack of Knowledge of Health Rights and the Services Offered at the Health Facility

The women lacked knowledge about their health rights. They had heard of or witnessed other women treated disrespectfully. Some participants had personally experienced rude and hurtful behavior from their healthcare provider. Women endured being shouted at by healthcare providers as normal behavior and did not complain about being treated respectfully. Instead, they understood that the healthcare provider shouted for their welfare and had no harmful intention.

The participants or their relatives were less knowledgeable about what services were offered (blood transfusion or cesarean section) in the basic birthing centers vs. referral hospitals. Therefore, in emergencies, the women were rushed to the nearby birthing center instead of being taken to the referral hospital.

*They would do cesarean section … maybe [smiles]. Now at that health facility, they do laparoscopy, they do all the things, that’s why I was taken there.*

*(P0012, 18 years, Janajati)*

Some local administrative offices even provided five times more incentive (about 50 USD) to the Nepalese government’s cash incentive scheme to encourage facility-based birth. Women were even uninformed that for postpartum women, public hospitals provide free meals. Some participants expected to receive hospital-like services from their local facilities. A majority expected to receive ultrasonography and laboratory services from the public health facilities, which can be provided only after laboratory personnel placement.

## 4. Discussions

This study explored women’s perspectives of good-quality care for the maternal services they had received. The overall participants’ perception of the quality of ANC and birthing services was satisfactory. Our participants lacked basic knowledge of their reproductive rights. They did not have many expectations either from the health facilities or from the healthcare providers. Less crowded and prompt services were among the expected changes. Additionally, participants expected ultrasonography, laboratory services, additional birthing beds, attendants’ beds, and free calcium supply.

Evidence suggests that the women’s satisfaction was associated with their characteristics, such as age, parity, ethnic group, education, and income level [32,33]. Younger-aged women had fewer expectations from health services [34,35], and less educated women were more satisfied with the quality of care than higher educated women [33,36], which must be similar to our younger and less educated women participants. Our study findings support this evidence that our participants were mostly young, less educated, and nullipara. Overall, they had fewer expectations of health services and healthcare providers and were thus satisfied without many expectations.

The literature supports that women with lower education assess healthcare providers based on their interpersonal relationships and skills rather than clinical competence [37,38]. Thaddeus and Maine explained that judgments of technical competence and the practitioners’ quality were beyond the interviewed women’s capacity [37], which was applicable in our study. Participants did not mention healthcare providers’ clinical skills. In contrast, in Bolivia, women raised concerns about healthcare providers’ technical capacity [39]. Positive personality characteristics—such as healthcare providers’ temperament and personality—were valued by women as quality care attributes [40,41,42]. Women appreciate kind, caring, culturally sensitive, flexible, and respectful behavior [43].

The health facilities’ cleanliness has been one of the most frequently mentioned reasons for dissatisfaction among clients in several studies [44,45,46]. However, in our study, participants generally found the health facility clean, except for one participant. Furthermore, participants mentioned that they did not use the toilets or drink water at the health facility, which is inconsistent with the overall duration that participants spent visiting the services. Possible reasons may be because about 25% of the rural population still practiced open defecation in Nepal [47], and hand pumps are used for drinking. Reasons for restraining from using toilets at public health facilities in India were dirty toilets without running water [45,48]. This might be another possible reason, but the participants laughed and did not answer. Women will continue using ANC if their expectations align with their beliefs and values [43]. Promptness of care is one of the key criteria of perceived good care [49]. Among improvements to health services, reducing waiting times was commonly suggested [18,39,40], similar to this study. In Riyadh, Tanzania, and Nigeria, women attending ANC services were dissatisfied mainly due to unnecessary delays by the healthcare providers, which they perceived as wasting their time [50,51,52]. Irrespective of education level, women were found impatient waiting at the clinic, emphasizing their preoccupations [53] and, in this study, domestic responsibilities. Women complained about the quality of care if the place was crowded in India and Cameroon [49,53]. Healthcare providers admitted that they could not provide quality health care to their patients and maintain privacy if the health facility was overcrowded [54]. Women find the consultation duration adequate, which they mentioned was 5 to 10 min in this study. On average, public clinics recorded 3.7 min, and private clinics recorded 6.6 min for ANC consultations in Gambia, which women felt was slightly inadequate [55].

Our participants lacked knowledge about their basic health rights to be treated with respect. They tried avoiding healthcare providers’ scolding by enduring birth pain and remaining silent. A higher percentage of our participants were young, belonged to minority ethnicities, and were from a marginalized population. Contrary to our study’s findings, previous studies showed that women felt entitled to receive respectful care without any abuse from healthcare providers [8,43,56]. In contrast, our participants showed a forgiving attitude towards the providers’ rude behavior, understanding that their intended motivation was for the women’s welfare. Similar to our study, in Nigeria, women perceived shouting as normative behavior and mentioned that healthcare providers did not intend to cause harm [57]. The World Health Organization recommends providing respectful maternity care, while disrespectful behavior affects consequences, such as non-utilization [58,59].

Our participants were unaware of schemes and services targeted toward them, such as free transportation schemes and free clothes distribution (warm bags), to increase facility-based births by the Nepalese government. In contrast, in other studies, women complained about the quality of the food provided at the hospital [8,60]. Surprisingly, women living in minority communities from high-income countries also remained unaware of the full range of hospital-provided services [61]. Our findings indicate that information regarding the targeted interventions and schemes has not reached the marginalized population.

The perceived quality of care can influence the decision of service utilization [9]. In this study, participants do not have many expectations from the healthcare providers and no clear perceptions on quality of care, and only three-fifths of participants have completed four ANCs and given institutional births. Herman et al. mention that patient satisfaction is a multidimensional concept built on a relationship between experiences and expectations [62]. Their needs and experiences drive participants’ utilization of services, whereas cognitively, participants have no clear quality construct. Women’s perceptions of the quality of care are determined by their own experiences or the experiences of people they know [3]. Women’s perceptions of the quality of healthcare facilities are also formed based on their female relatives’ recommendations and personal experiences, as found in this study. In Tanzania, Ethiopia, and Bolivia, community concerns about poor quality birthing services had discouraged women from utilizing birthing services [18,35,39]. Participants did not know what changes to expect to improve ANC and birthing services and did not have many suggestions concerning enhancing services. Similar to this finding, in India, women (especially nullipara women) expressed that they did not know what to expect in an institutional birth and therefore had no expectations [8]. Bleich et al. suggested that patients’ satisfaction with the healthcare system could be explained by their expectations [36]. Similarly, in another study, some women could not think of anything to suggest [40]. Immigrant women in the US felt that they were unqualified to offer opinions about the care they received and did not want to be perceived as disrespectful or critical by providing suggestions for improvement [63]. Women cannot demand quality in health services if they do not know what changes to expect. In contrast, in a qualitative study in Canada, participants knew what should be improved and mentioned that they wanted female-centered care, emotional support, and the need for psychosocial health assessment [41].

It must be addressed that younger and minority community women were overrepresented in this study. A different scenario should be expected if women of different characteristics, especially those from higher ethnic groups, parity, socioeconomic status, and education level, with exposure to health services were enrolled. In-depth interviews could not explore what good-quality maternal care meant for the women. Most participants repeatedly replied they “don’t know” or went silent on quality care expectations and suggestions to improve, so the author considered this as reaching the saturation point. The participants were approached only once for the interview; meeting them often could have allowed for exploring more information. The findings of this study are generalizable to the similar geographical and ethnic population within Nepal. They do not only represent the Morang population, although the study was limited to the Morang district.

## 5. Conclusions

Women of low socioeconomic status from marginalized ethnicities lacked knowledge of their basic reproductive rights, endured rude behavior from healthcare providers, and were less aware of their health status. Interpersonal communications were valued by our participants, and they preferred continuity of care. Women thought that the healthcare providers knew better than them about the quality of care. Women and their family members expected improvements in basic amenities and expected to receive all possible services from the nearby birthing centers. Women were unable to express what good-quality maternal health care meant for them. The patient’s perspective of quality of care is crucial to understand if women’s satisfaction and usage are to be measured. To increase the recommended four ANC visits and increase institutional birth targets, users should first understand the concept of quality to demand quality services. The findings have opened potential areas to guide future research among women regarding the various aspects of quality of care. Future studies could explore the association between women’s satisfaction and their characteristics, users’ knowledge on quality of care, and demand in quality of care. National and state governments should provide context-specific interventions and reach out to underprivileged and marginalized communities.

## Figures and Tables

**Table 1:** Initial context, subthemes, and themes for the quality of antenatal care and birthing services.

Theme	Sub-Theme	Initial Context
1. Women’s opinions and satisfactory factors of health services	1.1 Satisfaction with existing health services	Satisfaction with waiting duration before service and consultation time
		Patient flow and cleanliness
		Free medicine availability
	1.2 Preferred place of birth and less concern about health issues	Use both public and private services
		Less concerned about own health and less knowledge of services intended for them
2. Expectations of health facility and staff	2.1 Expectations of health staff	Staff friendly behavior and explanation
		Reduce negligence in ANC services
	2.2 Expected change in health services and facilities	Provision of ultrasonography, laboratory facilities, and additional beds
		Do not use toilets and water facilities at the health facility
		Unaffordable ambulance service
		Prompt service/less waiting time
3. A lack of suggestions to improve the quality of care	3.1 Cannot express the requirements of good-quality services	No further meaning of “good”-quality services
		Do not know what should be improved
	3.2 Lack of knowledge of health rights and the services offered at the health facility	Lack of knowledge on maternal services provided from a basic birthing center
		Expect facility like a referral center
